# *Eleutherococcus divaricatus* Fruits Decrease Hyaluronidase Activity in Blood Serum and Protect from Oxidative Damages in In Vitro Model

**DOI:** 10.3390/ijms25042033

**Published:** 2024-02-07

**Authors:** Jakub Gębalski, Milena Małkowska, Dorota Gawenda-Kempczyńska, Artur Słomka, Maciej Strzemski, Jan Styczyński, Daniel Załuski

**Affiliations:** 1 Department of Pharmaceutical Botany and Pharmacognosy, Ludwik Rydygier Collegium Medicum, Nicolaus Copernicus University, Marie Curie-Skłodowska 9, 85-094 Bydgoszcz, Poland; jakub.gebalski@cm.umk.pl (J.G.); milena.malkowska@cm.umk.pl (M.M.); dgawenda@cm.umk.pl (D.G.-K.); 2 Department of Pathophysiology, Ludwik Rydygier Collegium Medicum, Nicolaus Copernicus University, Marie Curie-Skłodowska 9, 85-094 Bydgoszcz, Poland; artur.slomka@cm.umk.pl; 3 Department of Analytical Chemistry, Medical University of Lublin, Chodźki 4a, 20-093 Lublin, Poland; maciej.strzemski@umlub.pl; 4 Department of Pediatric Hematology and Oncology, Ludwik Rydygier Collegium Medicum, Nicolaus Copernicus University, Marie Curie-Skłodowska 9, 85-094 Bydgoszcz, Poland; jstyczynski@cm.umk.pl

**Keywords:** antioxidants, anti-tyrosinase, anti-hyaluronidase, adaptogenic plants, phytochemicals, *Eleutherococcus*, leukemia

## Abstract

Fruits are very important dietary components and a source of biologically active compounds used in nutritional pharmacology. Particularly due to the presence of polyphenolic compounds, fruits play an important role in the prevention of diseases of civilization. Therefore, it is important to study the phytochemicals and biological activity of fruits, especially those with a long-standing use in ethnomedicine. In this study, we determined the chemical profile and biological activity of a methanolic extract of the *Eleutherococcus divaricatus* fruits. Amongst nine polyphenols studied, only chlorogenic acid, protocatechuic acid, and eleutheroside E have been detected. The extract showed a weak anti-hyaluronidase activity from bovine testicular in a range of 9.06–37.70% and quite high for human serum hyaluronidase from children diagnosed with acute leukemia in a range of 76–86%. A weak anti-tyrosinase activity was obtained in a range of 2.94–12.46%. Moreover, the extract showed antioxidant properties against DPPH radical, ABTS radical, and O_2_^•−^. In addition, the antioxidant activity of the extract was evaluated by FRAP assay and Fe^2+^ ion chelation assay. These preliminary studies partially justify the traditional use of the plant in inflammatory- and immune-related diseases, in which hyaluronidase and free radicals can participate. A difference in human serum hyaluronidase inhibition may result from the inter-patient variability. Regardless of that, the results mean that polyphenolic compounds may stimulate activity of hyaluronidase, as well as to protect cells from the oxidative damages. However, further studies in ex vivo and in vivo models are needed, including blood isolated from a larger number of patients.

## 1. Introduction

Plants are a crucial source of medicines for treating human diseases. For centuries, humans have used them for various disease entities without knowledge of the compounds that determine their biological activity [1,2,3]. The beginning of the “conscious” use of plants was the isolation of morphine by German apothecary Friedrich Sertürner in 1804 [4]. This research marked the beginning of a new era of work on the medicinal properties of plants, making it possible to treat diseases such as hypertension (reserpine), gout (colchicine), or cancer (paclitaxel, vincristine, and vinblastine) [5]. After a temporary decline in interest in plants due to the development of combinatorial chemistry in the search for drugs, the 21st century has seen a “renaissance” of drugs of natural origin [6]. This is related to two aspects: (1) plants are rich in secondary metabolites with a wide variety of chemical structures, which generate many pharmacophores with unique spatial structures, and (2) compounds of natural origin, unlike synthetic molecules, due to their biochemical functions in the plant, offer a good chance of potential interaction with proteins and the ability of intercellular permeation.

Leukemia is a blood-related malignancy characterized by transformed hematopoietic progenitors and diffuse infiltration of bone marrow. Globally, in 2020, leukemia accounted for approx. 2.5% and 3.1% of all new cancer incidence and mortality. As mentioned above, plants have had significant therapeutic potential for preventing or treating human diseases for thousands of years. Current advances in leukemia therapy promote the use of natural products to prevent the onset of cancer; as well, they are a source of new anticancer drugs. It is estimated that almost half of the drugs used currently in treating cancer are plant-based compounds and their derivatives. Vincristine and vinblastine are the first plant-derived anticancer drugs used to treat leukemia or other types of cancers. On the other hand, plant compounds are also utilized as chemo-preventive and supporting a standard treatment. However, in many cases, their use has not been scientifically proven and patients take a risk when they connect administration of such compounds with standard treatments. Additionally, some of them are ingredients of so-called nutriproducts, which are very popular products with multidirectional functions [7,8,9].

When looking for new plant-based drugs, we should follow the ethnopharmacological knowledge of our ancestors, who could treat people with great success. The *Araliaceae* family is an interesting group of plants which have been used in traditional healing systems to treat immune-related diseases [10,11]. This family includes, among others, the most important in traditional Chinese medicine (TCM), used for 4000 years, a “panacea” for many diseases, *Panax ginseng* C.A. Meyer. A well-known representative of this family, used as a substitute for *P. ginseng*, is *Eleutherococcus senticosus* (Rupr. and Maxim.) Maxim [12]. In both scenarios, the root serves as a valuable medicinal ingredient. However, the substantial expense involved in obtaining this raw material necessitates the exploration of alternative sources [13,14,15]. In our laboratory, research is under way on the fruits of *E. senticosus*, which, unlike the root, do not require a long maturation period (about five years). A little-known representative of the *Eleutherococcus* is *E. divaricatus* (Siebold and Zucc.) S. Y. Hu. *E. divaricatus*, also known as five fingers, a plant species that belongs to the *Araliaceae* family. It is native to Northeast Asia, including China, Korea, and Japan. This plant is known for its medicinal properties; it is believed to improve one’s immune system and overall health [16]. The roots of *E. divaricatus* are commonly used in traditional Chinese medicine as a tonic for the spleen and kidneys. It is also used to treat rheumatism, hypertension, and diabetes [17]. In addition, the plant’s leaves and berries can be used to make a tea which is said to have a calming effect on the body and mind [18]. The plant is also used extensively in the cosmetic industry due to its antioxidant and anti-inflammatory properties. It is believed to promote healthy skin and reduce the signs of aging [19,20,21,22]. However, the details on the mechanism of action are still uncompleted. Moreover, there is not much information about that species cultivated in Poland. For these reasons, we have hypothesized that the fruits contain phytochemicals that may be responsible for their anti-enzymatic and antioxidant activities. We chose hyaluronidase and tyrosinase as enzymes participating in many diseases, including leukemia and skin cancers. To obtain more reliable results, we studied both enzymes commercially available (hyaluronidase and tyrosinase) as well as serum hyaluronidase from children diagnosed with acute leukemia. In order to prove our hypothesis, the phytochemical techniques of in vitro and ex vivo biological tests were applied. 

## 2. Results and Discussion 

Fruits play a crucial role in the diet of both humans and animals. They are a vital source of vitamins, mineral salts, and fiber. It is interesting to note that nearly 3/4 of the food consumed by humans is estimated to be the nutrients of fruits and seeds on a dry weight basis. In addition to primary metabolites, fruits also contain an important group of compounds known as secondary metabolites, which are responsible for a wide range of health-promoting effects [23]. Among these, polyphenolic compounds present in fruits exhibit a broad spectrum of activity. These insights highlight the immense nutritional and health benefits offered by fruits.

**Figure 1:**
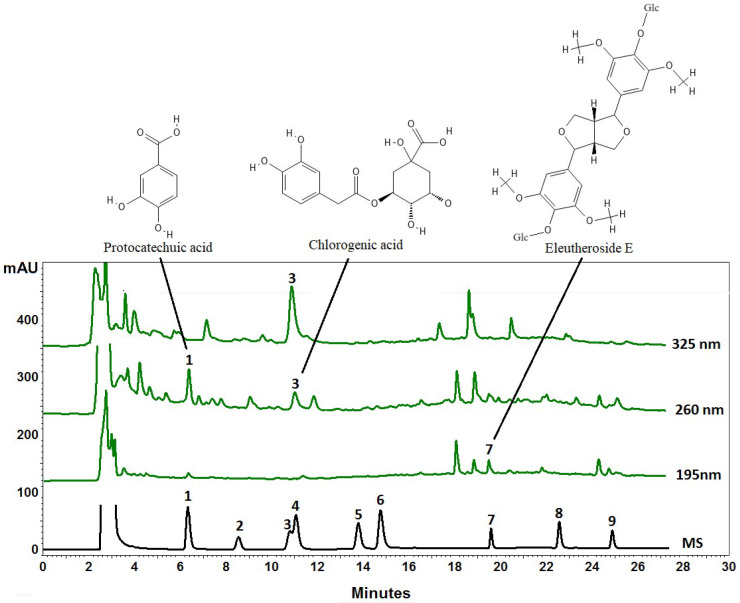
An exemplary HPLC chromatogram of *Eleutherococcus divaricatus* fruits extract (green line) and reference compounds (black line): 1-protocatechuic acid, 2-eleutheroside B, 3-chlorogenic acid, 4-*p*-hydroxybenzoic acid, 5-vanillic acid, 6-caffeic acid, 7-eleutheroside E, 8-ferulic acid, and 9-eleutheroside E1. Conditions: RP18 reversed-phase column Kinetex at 25 °C, a mixture of acetonitrile (solvent A) and water (solvent B), both acidified with 0.025% of trifluoroacetic acid, were used as the mobile phase. The compounds were separated by gradient elution with program: 0.0–8.0 min A 10%, B 90%; 8.1–18.0 min A 10–20%, B 90–80%; 18.1–28.0 min A 20%, B 80%; 28.1–35.0 min A 20–25%, B 80–75%; and 35.1–40.0 min A 25%, B 75%. Flow rate was 1.0 mL/min.

Table 1 presents the quantitative results for eleutherosides and phenolic acids. Figure 1 presents a chromatogram for eleutherosides and phenolic acids.

The extraction of the fruits resulted in 22.5% dry extract yield and the total polyphenol content was found to be at a level of 14.61 mg GAE/g. However, using the spectrophotometric methods, flavonoids, phenolic acids, and tannins were not detected.

The literature provides limited information on the chemical composition of *E. divaricatus* fruits. The low polyphenol content could be attributed to the solvent and extraction method used. Załuski et al. highlighted the impact of the solvent on the total polyphenol content of extracts prepared from *E. divaricatus* fruits, reporting 52.03 ± 0.5 mg GAE/g for 75% EtOH (accelerated solvent extraction) and 41.1 ± 0.5 mg GAE/g for infusion (95 °C distilled water). Additionally, the fruits were collected in 2016 and the differences may result from a seasonal change [24]. In a previous study, the contents of polyphenols, flavonoids, and phenolic acids in the intractum made from other *Eleutherococcus* species, i.e., *E. senticosus* fruits, were found to be 1.02 ± 0.04 mg GAE/g DW, 0.1 ± 0.05 mg QE/g DW, and 0.30 ± 0.07 mg CAE/g DW, respectively [25]. An investigation of 75% ethanol extracts made from fresh and dried fruits of *E. senticosus* and *E. henryi* revealed no statistically significant differences between the polyphenol levels (for *E. senticosus*—fresh dried 4110 mg GAE/100 g, storage 3850 mg GAE/100 g; and for *E. henryi*—fresh dried 4350 mg GAE/100 g, storage 4140 mg GAE/100 g) [26]. Conversely, the methanolic extract made from *E. senticosus* fruits (1:10 dry weight material to MeOH) contained 229.83 ± 9.34 mg GAE/g of polyphenols [27]. Similar results were obtained in a study examining polyphenol levels from fresh fruits of *E. divaricatus*. The total polyphenol content (TPC) for other species (*E. senticosus*, *E. gracilistylus*, *E. sessiliflorus*, *E. henryi*, and *E. setchuensis*) ranged from 6.9 ± 0.01 to 19.7 ± 0.01 mg/g [28]. In those cases, it should be noted that the content of phytochemicals in plants is dependent on a few factors, including, among others, the seasonal and weather conditions. Therefore, a quality control is a needed process at every step of plant raw material processing.

In the next step, the contents of phenolic acids (protocatechuic acid, chlorogenic acid, *p*-hydroxybenzoic acid, vanillic acid, caffeic acid, and ferulic acid) and eleutherosides (B, E, and E1) were examined. Out of all tested compounds, only chlorogenic acid (0.13 ± 0.01 mg/g extract ± SD), protocatechuic acid (1.47 ± 0.10 mg/g extract ± SD), and eleutheroside E (0.23 ± 0.01 mg/g extract ± SD) have been detected. Eleutheroside B and E have also been detected in the fruits of this species by Kim et al., in the amounts of 1.06 and 7.08 μg/mg, respectively [29]. Comparing these results with the results obtained for the fruits of *E. senticosus*, used very often as a model species for that genus, Bączek discovered eleutheroside B (0.356 mg/g) and E (0.298 mg/g) in an ethanol extract of *E. senticosus* fruits [30]. Our previous investigation also confirmed their presence in *E. senticosus* (0.66 and 0.74 mg/gDW, respectively) [31]. Taking into consideration the phenolic acids, an extract from *E. senticosus* fruits contained protocatechuic acid, 4-OH-benzoic acid, vanillic acid, caffeic acid, ferulic acid, and rosmarinic acid [32]. The extract from *E. senticosus* fruits contained 4.1 mg/g of chlorogenic acid and 0.84 mg/g of rosmarinic acid. These compounds were present in four-year-old raw materials, while two-year-old and three-year-old raw materials did not contain these substances [30]. The study by Załuski et al. identified protocatechuic acid (0.45 mg/gDW), 4-OH-benzoic acid (2.0 mg/gDW), vanillic acid (4.2 mg/gDW), *trans*-caffeic acid (41.2 mg/gDW), and *trans*-ferulic acid (3.6 mg/gDW) in the *intractum* from the *E. senticosus* fruits [33]. The varying content of polyphenolic compounds can be attributed to different extraction methods, the solvents used in the study, and the location where the raw material was harvested.

The antioxidant capacity of a methanolic extract from *E. divaricatus* fruits was evaluated using several methods. The application of multiple methods enabled a more precise estimation of the extracts’ antioxidant properties. The extract’s activity was assessed against DPPH^•^, ABTS^+^, and O_2_^•−^. This marks the first time, to our knowledge, that the O_2_^•−^ scavenging capacity of an extract made from fruits of this species has been evaluated. The antioxidant activity was also assessed using the Fe^2+^ ion chlorination test and the FRAP method. The results, presented in Table 1, showed that the extract exhibited moderate antioxidant properties. In the DPPH and ABTS assays, the IC_50_ values were 1.36 and 0.28 mg/mL, respectively. The activity against superoxide radical was 1.51 mg/mL. The IC_50_ for chelating Fe^2+^ ions were 1.45 mg/mL. In the FRAP test at concentrations of 1 mg/mL and 0.1 mg/mL, the values were 6.01 and 5.02 mg Trolox/g, respectively, in comparison to BHA, 28.91 and 12.83 mg Trolox/g.

The antioxidant properties of the fresh *E. divaricatus* fruits, expressed as the EC_50_ value, were evaluated in a study involving five *Eleutherococcus* species. The fruits of *E. divaricatus* showed the highest activity (2.7 mg/mL) in the linolenic acid oxidation inhibition test. The activity of other species (*E. setchuensis*, *E. senticosus*, *E. gracilistylus*, and *E. henryi*) ranged from 3.4–4.9 mg/mL. In this study the chelating capacity of Fe^2+^ ion was also evaluated, with the EC_50_ value obtained for all extracts equal 0.4 mg/mL. The EC_50_ of *E. divericatus* fruit extract against the DPPH radical was 86.2 ± 2.0 mg/mL. The range of activity for other species was from 4.5 ± 0.2 to 63.4 ± 0.5 [28]. Załuski et al. reported on an anti-DPPH activity of the *E. senticosus* and *E. henryi* ethanol fruits extracts with the IC_50_ in a range of 0.1–0.29 mg/mL [26]. The acidified 80% MeOH extract of *E. senticosus* fruits exhibited stronger antioxidant properties against DPPH, ABTS, and hydroxyl radicals (IC_50_ values of 11.2, 4.3, 14.5 μg/mL, respectively) when compared to cyanidin-3-O-(2″-O-xylosyl)-glucoside isolated from this extract (IC_50_ values of 85.2, 43.7, and 126.6 μg/mL, respectively) [34]. Kim et al. determined the antioxidant activity of aqueous extracts made from *E. senticosus* and *E. koreanum* fruits. *E. koreanum* showed strong antioxidant properties in DPPH, ABTS, FRAP, and ORAC tests compared to extracts made from the roots, leaves, and stem of this plant. In contrast, for *E. senticosus*, the antioxidant activity in these tests was significantly weaker [29].

For centuries, plants have been used as medicines to treat human diseases. The use of plants as therapeutic agents was made possible by the presence of a variety of chemical compounds characterized by a diverse biological activity. In the above work, we tested how an extract made from the fruits of *E. divaricatus* affects human serum hyaluronidase from children diagnosed with acute leukemia (AL), bovine hyaluronidase, and fungal tyrosinase. The results are shown in Table 2 and Table 3. It is well known that the activity of hyaluronidase increases in many diseases, e.g., in leukemia, skin conditions, or GI tract diseases. Simultaneously, in this case, it should be noted that the overactivity plays both positive and negative roles, with the overweighting of the negative one. The elevated amount in serum has been detected in the case of hepatitis C and pancreaticocutaneous fistula. In Crohn’s disease, the elevated hyaluronidase amount was detected in colonic fibroblasts. The latest reports provide information about the subcutaneous injection of rituximab and hyaluronidase for the treatment of adults with follicular lymphoma (FL), diffuse large B-cell lymphoma (DLBCL), or chronic lymphocytic leukemia (CLL). In this case, hyaluronidase serves as a spreading factor and is inactivated in skin shortly after injection [35].

To assess whether the fruits might regulate the hyaluronidase activity, we used serum from patients diagnosed with acute leukemia (AL) before starting treatment. Firstly, the activity of hyaluronidase was established; next, the extract in a dose 100 μg was added to each serum sample. The dose 100 μg has been previously selected in research on the immunostimulative activity of the fruits of *Eleutherococcus senticosus* and eleutherosides, another species with the *E. divaricatus*-like activity and which is used as a model species in our long-standing research. Table 3 presents the findings of the study on the effectiveness of the extract against human serum hyaluronidase. The extract exhibited an activity range of 76.46% to 86.13% when it is compared to escin (56.17% to 95.62%, respectively). In the case of bovine hyaluronidase, the methanolic extract showed moderate activity (9.06–37.70%); similar results were obtained for escin 4.15–58.96%. Moreover, a weak anti-tyrosinase activity was obtained in a range of 2.94–12.46% and strong for kojic acid 14.32–99.39%. To our knowledge, this is the first report on the impact of *Eleutherococcus* fruits extract on human serum hyaluronidase.

The IC_50_ for extracts made from the fruits of *E. senticosus* and *E. divaricatus* against bovine hyaluronidase (hyal) ranged from 0.58 to 0.87 mg/mL [30]. In a test of the activity of *E. senticosus* fruit *intractum* against hyal, the IC_50_ was 217.44 ± 10.72 μg/mL, against tyrosinase was equal 586.83 ± 2.36 μg/mL [29]. The activity of methanol extracts made from the roots of *E. gracilistylus*, *E. divaricatus*, *E. senticosus*, *E. henryi,* and *E. sessiliflorus* was 19.6–32% against hyal [36]. In the other study, autumn leaves inhibited hyal stronger when compared to spring leaves (74.3 and 33%, respectively) [37].

An inhibition of hyaluronidase and tyrosinase have been described by many researchers who tested both the extracts and isolated compounds [38,39,40]. There are many factors the inhibition is reliant on. It makes the comparison of results, sometimes, impossible. Additionally, there is a lack of standardized units of enzyme activity and in different investigations different units are used. In many publications, no units’ activity are provided, even for those enzymes commercially available. Another problem is a lack of an absorbance assay for the extracts alone. The extracts and some isolated plant-based compounds contain dyes or are dyes themselves; finally, they can strengthen the absorbance, giving a false positive result. In the case of oxidoreductases, some compounds might act also as substrates and inhibitors or just like substrates, especially when studied in an isolated form [41]. In this case, a series of other tests are needed, like NMR titration, and also establishing what are the most reliable tests from a pharmacological point of view, including cells or animal model tests.

## 3. Materials and Methods

### 3.1. Chemicals and Reagents

Nitrotetrazolium blue chloride (NBT), xanthine, xanthine oxidase, 2,2-diphenyl-1-picrylhydrazyl (DPPH), 2,2′-azinobis-(3-ethylbenzthiazoline-6-sulfonic acid) (ABTS), potassium persulfate, 3-(2-Pyridyl)-5,6-diphenyl-1,2,4-triazine-p,p′-disulfonic acid monosodium salt hydrate (ferrozine), iron (II) chloride tetrahydrate (FeCl_2_ × 4H_2_O, 1,3,5-Tri(2-pyridyl)-2,4,6-triazine (TPTZ), iron (III) chloride (FeCl_3_), aluminum chloride (AlCl_3_), potassium acetate, Folin−Ciocalteu reagent, sodium nitrite, sodium molybdate, 6-hydroxy-2,5,7,8-tetramethylchroman-2-carboxylic acid (Trolox), ascorbic acid, 2(3)-t-Butylhydroquinone monomethyl ether (BHA), 2(3)-t-Butyl-4-hydroxyanisole, hyaluronic acid (IV), escin, hyaluronidase from bovine testes, hexadecyltrimethylammonium bromide (CTAB), L-tyrosine, koji acid, and tyrosine from mushrooms were purchased from Sigma-Aldrich Corp. (St. Louis, MO, USA). The standards of eleutheroside B ≥98.0% (HPLC), eleutheroside E ≥98.0% (HPLC), eleutheroside E1 ≥98.0% (HPLC), protocatechuic acid ≥97%, p-hydroxybenzoic acid 99%, vanillic acid ≥97%, caffeic acid ≥98%, and ferulic acid ≥99% were also purchased from Sigma-Aldrich. Solvents used for extraction were purchased from Avantor Performance Materials (Gliwice, Poland).

### 3.2. Preparation of Extract 

The fruits were collected from the Arboretum SGGW in Rogów, Poland, in 2021 and authenticated by Prof. D. Załuski. The fresh fruits were air-dried at room temperature and macerated with a 75% methanol solution (15 g/150 mL). The extract was then subjected to ultrasound treatment for 15 min and repeated three times. The resulting extract was evaporated and stored in a refrigerator at 2 °C. The extraction yield was calculated based on the dry weight of the extract (%).

### 3.3. Phytochemical Panel

Spectrophotometric and chromatographic methods were used to determine the quantitative and qualitative composition of the extract. The methods used are described in more detail in a previous paper.

#### 3.3.1. Chemical Composition 

##### Determination of Total Phenolic Content (TPC)

A modified version of the Folin–Ciocalteu method was used to determine the total phenolic content [42]. The extract (1 mg/mL in MeOH) was mixed with Folin−Ciocalteu reagent (diluted in pure water, 1:3) at a ratio of 1:1 each. The mixture was then incubated for 5 min after adding distilled water. Sodium carbonate (10%) solution was added and the mixture was incubated in the dark at room temperature for an hour. The absorbance was measured at 750 nm. The TPC results were expressed in milligrams of gallic acid (GA) equivalents (GAE) per gram of the sample (mg GAE/g sample).

##### Determination of Total Phenolic Acid Content (TTC)

To determine total tannin content, we used the Zhu method with slight modification [43]. The polyvinylpolypyrrolidone (PVPP) was used to precipitate the tannins. Quickly, to 1 mL of extract in methanol, we added 1 mL of PVPP (0.5%). The samples underwent a thorough mixing process using a vortex. Following this, they were subjected to a low-temperature incubation at 4 °C for a duration of 10 min. The final step in the procedure involved centrifugation at a speed of 5000 rpm, lasting for 5 min. The supernatant was used to determine the total phenolic content by the methods described above. The TTC results were expressed in milligrams of tannic acid (TA) equivalents (TAE) per gram of the sample (mg TAE/g sample).

##### Determination of Total Flavonoids Content (TFC) 

The total flavonoid content was determined using a method that involved the reaction between AlCl_3_ and flavonoids [44]. In short, extract (1 mg/mL in MeOH) and EtOH was mixed and, then, aluminum chloride (10%) and potassium acetate (1 M) were added. The mixture was incubated for 30 min after adding distilled water. The absorbance was measured at 510 nm and the results of TFC were expressed in milligrams of quercetin equivalents (QE) per gram of the sample (mg QE/g sample).

##### Determination of Total Phenolic Acid Content (TPAC)

The method outlined in Polish Pharmacopeia VI was followed to determine the total phenolic acid content [45]. The extract (1 mg/mL in MeOH) was mixed with distilled water, HCl (0.5 M), and Arnov’s reagent (10.0 g of sodium molybdate and 10.0 g of sodium nitrite in 100 mL distilled water). Solution of NaOH (1M) was added and the mixture was immediately measured at 492 nm. The TPAC results were expressed as milligrams of caffeic acid (CA) equivalents (CAE) per gram of the sample (mg CAE/g sample). 

#### 3.3.2. HPLC-PDA-Based Metabolomic Profiling of Phenolic Compounds in the Extract

The EliteLaChrom chromatograph with PDA detector and EZChrom Elite software 3.2.0 (Merck, Darmstadt, Germany) was used to perform the analyses. The gradient chromatographic system used in the experiment consisted of an RP18 reversed-phase column Kinetex (Phenomenex, Torrance, CA, USA) measuring 25 cm × 4.6 mm i.d., with a particle size of 5 μm, maintained at a temperature of 25 °C. A mobile phase comprising acetonitrile (solvent A) and water (solvent B) was used, containing 0.025% trifluoroacetic acid. The compounds were separated by gradient elution using a program that included various percentages of solvents A and B over a period. The flow rate was 1.0 mL/min and data were collected between 190 and 400 nm. The identity of compounds was established by comparing their retention times and UV spectra with corresponding standards. Quantitative analysis was performed at specific wavelengths for each compound (260 nm for protocatechuic acid, 325 nm for chlorogenic acid, and 195 nm for eleutheroside E).

### 3.4. Enzymatic Panel

#### 3.4.1. Bovine Hyaluronidase Inhibition Assay

Bovine hyaluronidase inhibitor assays were performed in 96-well plates using a modified method described by Di Ferrante [46] and Studzińska-Sroka [47]. The precipitation of the undigested hyaluronic acid with cetyltrimethylammonium bromide (CTAB) was determined as the activity of the compounds/extracts. Three concentrations of extracts 0.1, 1.0, and 10 mg/mL (final concentration in well: 1.0, 10, and 100 μg/300 μL), acetate buffer (pH = 5.35), incubation buffer (pH = 5.35, 0.01% BSA, and 0.45% NaCl) and enzyme (30 U/mL in incubation buffer) were mixed. The mixture was incubated at 37 °C for 10 min. Then, hyaluronic acid solution (0.3 mg/mL in acetate buffer pH = 5.35) was added. The plates were further incubated for 45 min at 37 °C. After incubation, undigested HA was precipitated by adding 2.5% of CTAB. The plates were kept at 25 °C for 10 min. The intensity of complex formation was measured at 600 nm. The presence of inhibition was determined by measuring the absorbance of the solution without inhibitor (A_C_) and enzyme (A_T_). All samples were tested in triplicate. The hyaluronidase inhibition was calculated using the following equation and escin was used as a standard:

$${\%}_{INH}=\left(\frac{A_{S}-A_{C}}{A_{T}-A_{C}}\right)\times 100\%$$

A_S_—absorbance of the HA + sample + enzyme

A_C_—absorbance of the HA + enzyme

A_T_—absorbance of the HA + sample.

#### 3.4.2. Human Serum Hyaluronidase 

Three children with a median age of 7 years (range, 6–8 years), diagnosed with acute leukemia (AL) before starting treatment, were included in the study. The patients were diagnosed at the Department of Pediatric Hematology and Oncology (Jurasz University Hospital, Bydgoszcz, Poland) in 2019–2020. Venous blood was collected from each child under fasting conditions and placed into serum tubes (Becton Dickinson, Franklin Lakes, NJ, USA). Blood samples were allowed to clot for 30 min at room temperature and then were centrifuged for 20 min at 2000× *g* at room temperature. They were collected and stored at −80 °C until analyses. The local bioethics committee approved the study (608/2019). It was carried out in accordance with the Declaration of Helsinki.

##### Level of Human Serum Hyaluronidase 

The commercially available kit (LS-F6310 Human Hyaluronidase (Sandwich ELISA) ELISA Kit) was utilized to measure the concentration of human hyaluronidase in serum. This kit operates on the sandwich assay principle and is capable of detecting hyaluronidase levels down to 0.115 nanograms per milliliter. 

##### Human Serum Hyaluronidase Inhibition Assay

The inhibition of human serum hyaluronidase was evaluated using modified methods [25]. The activity of the compounds/extracts was determined by precipitating the undigested hyaluronic acid with cetyltrimethylammonium bromide (CTAB). Briefly, 10 μL of extract 10 mg/mL (final concentration in well 100 µg/300 μL) and 50 μL of serum was incubated at 37 °C for 15 min. Subsequently, a 40 μL of solution of hyaluronic acid (0.3 mg/mL in acetate buffer with pH = 5.35) was added. The plates were incubated for an additional 45 min at 37 °C. After incubation, undigested HA was precipitated by adding 2.5% CTAB. The plates were shaken out at 25 °C for 10 min. The intensity of complex formation was measured at a wavelength of 600 nm. All samples were tested in triplicate. The inhibition of hyaluronidase was calculated using a specific equation, with escin used as a standard.

$${\%}_{INH}=\left(\frac{A_{S}-A_{C}}{A_{T}-A_{C}}\right)\times 100\%$$

A_S_—absorbance of the HA + sample + enzyme

A_C_—absorbance of the HA + enzyme

A_T_—absorbance of the HA + sample.

#### 3.4.3. Tyrosinase Inhibitor Assays

Tyrosinase inhibitor assays were performed in 96-well plates according to a modified method [25,48]. Conversion of L-tyrosine to L-DOPA and L-DOPA to DOPA-quinone, accompanied by the browning of the solution, is catalyzed by tyrosinase enzyme. Briefly, 10 μL of three concentrations of extracts 0.1, 1.0, and 10 mg/mL sample (final concentrations in well: 1.0, 10, and 100 μg/200 μL) and 150 μL of phosphoric buffer with mushroom tyrosinase (pH = 6.88, 100 U/mL) were mixed and incubated for 10 min at room temperature. In addition, a control without inhibitor was prepared (A_C_). After incubation, L-tyrosine (0.3 mg/mL) was added to each well and the absorbance was measured at 492 nm (kinetic model, every 5 min). Next, two time points (t_1_ and t_2_) were selected in the linear range of the graph. All samples were tested in triplicate. The tyrosinase inhibition was calculated using the following equation and kojic acid was used as a standard:

$${\%}_{INH}=\left(\frac{A_{S}-A_{C}}{A_{C}}\right)\times 100\%$$

A_S_—the difference in absorbance between times t_2_ and t_1_ for sample,

A_C_—the difference in absorbance between times t_2_ and t_1_ for positive control.

All analyses were performed in triplicate. 

### 3.5. Antioxidant Panel 

#### 3.5.1. ABTS Free Radical Scavenging Activity

The method described by Wu et al. was followed to test for ABTS free radical scavenging [49]. A working solution of ABTS+ was prepared by mixing 10 mL of ABTS (7 mM in H_2_O) with 10 mL of potassium persulfate (2.45 mM in H_2_O), which was left to incubate in the dark for 12 h. The ABTS^+^ solution was diluted with water to achieve an absorbance of 0.700 ± 0.03 at 405 nm. Next, extracts at concentrations of 0.1 mg/mL, 1 mg/mL, and 10 mg/mL (final concentration in well: 1, 10, and 100 μg/200 μL) were mixed with 190 μL of the ABTS^+^ solution and incubated for 30 min. After incubation, the absorbance at 405 nm was measured. BHA was used as control. The antioxidant activity was calculated using the provided equation.

$${\%}_{INH}=\left(\frac{A_{S}-A_{C}}{A_{C}}\right)\times 100\%$$

A_S_—the absorbance for sample + ABTS

A_C_—the absorbance without sample + ABTS.

#### 3.5.2. DPPH Free Radical Scavenging Activity

The method for testing DPPH free radical scavenging was followed as outlined by Naseer et al. [50]. A working DPPH^•^ solution was created by dissolving 24 mg of DPPH in 100 mL of distilled water. The solution was then diluted with methanol until an absorbance of 0.900 ± 0.03 at 515 nm was reached. Next, extracts at concentrations of 0.1 mg/mL, 1 mg/mL, and 10 mg/mL (final concentration in well: 1, 10, and 100 μg/200 μL) were mixed with 190 μL of the DPPH^•^ solution and incubated for 60 min. The absorbance at 515 nm was measured after incubation. BHA was used as control. The antioxidant activity was then calculated using the given equation.

$${\%}_{INH}=\left(\frac{A_{S}-A_{C}}{A_{C}}\right)\times 100\%$$

A_S_—the absorbance for sample + DPPH

A_C_—the absorbance without sample + DPPH.

#### 3.5.3. Ferric-Ion-Reducing Antioxidant Power (FRAP) Assay

The FRAP assay was conducted by mixing extracts of 0.1 and 1.0 mg/mL (at final concentrations of 1 and 10 μg/300 μL) with 290 μL of a working solution consisting of acetate buffer (15 mL), TPTZ solution (1.5 mL), and FeCl_3_ × 4H_2_O (1.5 mL). The mixture was then incubated for 30 min before measuring the absorbance at 593 nm. Trolox and BHA were used as control. The results of the FRAP assay were expressed in milligrams of Trolox per gram of the sample (mg Trolox/g sample) [51].

#### 3.5.4. Iron (II) Ion Chelation Assay

Li et al. method was employed to determine the ion chelation assay [52]. Firstly, extracts of 0.1 and 1.0 mg/mL (with final concentrations of 10 and 100 μg/260 μL in the well) were mixed with MeOH and FeCl_2_ (2 mM). Then, ferrozine (5 mM) was added. After incubation, the absorbance at 510 nm was measured. The chelation was calculated using the following equation, with EDTA being used as a positive control.

$${\%}_{chel.}=\left(1-\frac{A_{S}}{A_{C}}\right)\times 100\%$$

A_S_—the absorbance for sample + ferrozine + FeCl_2_

A_C_—the absorbance without sample + ferrozine + FeCl_2_.

#### 3.5.5. O_2_^•–^ Scavenging Capacity Assay

Scavenging of the superoxide anion was examined by using a xanthine–xanthine oxidase system with the nitro blue tetrazolium chloride (NBT) described by Choi et al. [53]. Briefly, 50 μL of extract at concentration 10, 1, and 0.1 mg/mL (500, 50, and 5 μg/200 μL), 100 µL of a solution of xanthine with NBT (1:1 (*v*/*v*); 0.4 mM and 0.24 mM, respectively) and 50 µL of a mixture of xanthine oxidase (10 mU). After 20 min of incubation at 37 °C, the absorbance was determined at 560 nm. All reagents were dissolved in PBS. As a positive control, ascorbic acid was used. 

### 3.6. Statistical Analysis

Statistically significant differences between the extracts and control substances were calculated based on the obtained percentage values of anti-enzymatic or antioxidant activity. None of the samples exhibited a normal distribution; therefore, non-parametric Kruskal–Wallis tests were used.

## 4. Conclusions

It is clearly seen that there are compounds in the extract which have a significant anti-hyaluronidase activity with the mean value of 82.51% by a patient’s group. We can suggest that chlorogenic acid, because of its already confirmed anti-hyaluronidase activity, may represent these compounds [54,55,56].

Overexpression of hyaluronidases causes increased cell proliferation; therefore, their inhibition is a new strategy for the treatment of several diseases, as well as neoplastic inflammatory-related diseases [57,58,59,60,61]. Considering our results, research on synergism and antagonism with drugs used in the treatment of the above-mentioned diseases are needed, including blood samples isolated from a larger number of patients.

Based on these findings, it can be concluded that *E. divaricatus* fruits could strengthen a general vitality of the body. And the results meet criteria about plant-based compounds, which should act rather gently and be used for a longer time to be effective.

## Figures and Tables

**Table 1:** Antioxidant activity of *E. divaricatus* fruits. The results are presented as IC_50_ (mg/mL ± SD).

	ABTS *	DPPH **	CA **	O_2_^•−^ **
Extract	0.28 ± 0.01	1.30 ± 0.01	1.45 ± 0.11	1.51 ± 0.11
BHA	0.0025 ± 0.00	0.40 ± 0.01	-	-
AA	-	-	-	0.05 ± 0.00
EDTA	-	-	0.19 ± 0.00	-

BHA—butylhydroxyanisole, AA—ascorbic acid, EDTA—ethylenediaminetetraacetic acid, and CA—ion chelation. * *p* < 0.05 and ** *p* < 0.01, in the table column, indicate statistically significant differences (Kruskal–Wallis test).

**Table 2:** Inhibition of *E. divaricatus* fruits towards serum human hyaluronidase from children diagnosed with acute leukemia (AL) before starting treatment (%). The results are presented for an extract concentration of 100 μg and expressed as %.

Patient’s Age	Serum Hyal (U/mL)	*E. divaricatus*	Escin
		(%)	(%)
6	130.29	84.94 ± 10.28	56.17 ± 18.95
8	38.60	86.13 ± 9.29	95.62 ± 24.07
7	136.55	76.46 ± 0.18	61.35 ± 11.74
Means	101.81	82.51	71.04

The Kruskal–Wallis test revealed statistically significant differences between the effects of the extract and escin (*p* < 0.05), regardless of the patient. However, subsequent post hoc tests did not show statistically significant differences between the extract and escin within individual patients.

**Table 3:** Inhibition of *E. divaricatus* fruits towards bovine hyaluronidase and tyrosinase. The results are presented as % of inhibition and IC_50_ (mg/mL ± SD).

		Hyal *		Tyr *	
	Concentration (μg/300 μL)	(%)	(IC_50_)	(%)	(IC_50_)
Extract	100 10 1	37.70 ± 3.09 9.06 ± 1.54 0.00 ± 0.00	0.45 ± 0.04	12.46 ± 2.30 6.02 ± 3.02 2.94 ± 2.41	2.67 ± 0.05
Escin	100 10 1	58.96 ± 1.69 9.88 ± 2.05 4.15 ± 1.62	0.28 ± 0.01		
Kojic acid	100 10 1			99.39 ± 0.33 34.04 ± 1.60 14.32 ± 1.90	0.027 ± 0.00

Hyal—hyaluronidase, Tyr—tyrosinase. * *p* < 0.01, in the table column, indicates statistically significant differences (Kruskal–Wallis test).

## Data Availability

The data presented in this study are available on request from the corresponding author.
